# Cross‐talk between circadian clocks, sleep‐wake cycles, and metabolic networks: Dispelling the darkness

**DOI:** 10.1002/bies.201500056

**Published:** 2016-02-11

**Authors:** Sandipan Ray, Akhilesh B. Reddy

**Affiliations:** ^1^ Department of Clinical NeurosciencesUniversity of Cambridge Metabolic Research LaboratoriesNational Institutes of Health Biomedical Research CentreWellcome Trust‐Medical Research Council Institute of Metabolic ScienceUniversity of CambridgeAddenbrooke's HospitalCambridgeUnited Kingdom

**Keywords:** circadian rhythms, metabolic networks, non‐transcriptional oscillator, peroxiredoxin, redox regulation, sleep‐wake cycle, systems biology

## Abstract

Integration of knowledge concerning circadian rhythms, metabolic networks, and sleep‐wake cycles is imperative for unraveling the mysteries of biological cycles and their underlying mechanisms. During the last decade, enormous progress in circadian biology research has provided a plethora of new insights into the molecular architecture of circadian clocks. However, the recent identification of autonomous redox oscillations in cells has expanded our view of the clockwork beyond conventional transcription/translation feedback loop models, which have been dominant since the first circadian period mutants were identified in fruit fly. Consequently, non‐transcriptional timekeeping mechanisms have been proposed, and the antioxidant peroxiredoxin proteins have been identified as conserved markers for 24‐hour rhythms. Here, we review recent advances in our understanding of interdependencies amongst circadian rhythms, sleep homeostasis, redox cycles, and other cellular metabolic networks. We speculate that systems‐level investigations implementing integrated multi‐omics approaches could provide novel mechanistic insights into the connectivity between daily cycles and metabolic systems.

**Abbreviations**
CLOCK: circadian locomotor output cycles kaput
EEG: electroencephalograms
NTO: non‐transcriptional oscillator
PRX: peroxiredoxin
RBC: red blood cell
SCN: suprachiasmatic nucleus
TTFL: transcription‐translation feedback loop

## Introduction

Circadian (approx. 24 hour) clocks are believed to exist at almost all levels of life and play a key role in the maintenance of physiological and behavioral processes in accordance with the day/night cycle 1, 2. Similarly, sleep is thought to be a critical process in higher organisms 3. However, our view of sleep as being a product of the brain, or even neuronal populations, may obscure underlying principles and function of sleep. For example, in mammals, sleep is invariably measured using electroencephalograms (EEGs), which may not be the best way to characterize or quantify sleep in the molecular era. This is particularly pertinent in organisms such as the fruit fly, in which electrical recordings are correlated with behavioral activity 4, but not yet clearly to sleep‐wake cycles. However, it may equally be the case in mammals, in which wide ranging changes in gene expression are seen in the livers of sleep‐deprived mice 5, and in the responsiveness of adipose tissue to insulin signaling in humans under sleep restriction 6. The circadian clockwork and sleep‐wake cycles closely interact with each other, which is most obviously seen by the gating of sleep at distinct phases of the 24‐hour cycle. Thus, artificially separating these cycles may obscure underlying principles that unite both phenomena.

Adequate sleep is an essential requirement for health. However, a significant proportion of the adult population suffers from trouble sleeping at night, and staying awake during the daytime, most likely due to the aberrations in the switching mechanism that controls transitions between wake and sleep 7. Understanding the neurobiological mechanisms underlying the circadian system and sleep, and their interconnectivity, thus has profound implications for translational healthcare research, since circadian misalignment or aberrations in sleep homeostasis through old age, neurological diseases, and even shift work, are a rising cause of considerable morbidity 8, 9, 10, 11. Moreover, several studies indicate an intimate association of circadian dysfunction and sleep disruption with different human diseases including cancers, heart disease, diabetes, metabolic, vascular, and mental disorders (reviewed in 12, 13, 14).

**Figure 1:**
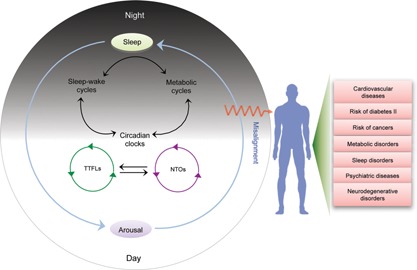
Interplay among circadian rhythmicity, metabolic cascades and sleep homeostasis: some opaque zones in circadian biology research. Cross‐talk among the circadian oscillators, sleep‐wake cycle and metabolic oscillations are important components of daily biological time‐keeping mechanisms. However, the precise mechanisms by which sleep‐wake centers, circadian clocks, and metabolic pathways communicate with each other have not been clearly demarcated. Interaction between non‐transcriptional oscillators (NTOs) and transcription‐translation feedback loop (TTFL)‐based oscillators is also largely unclear. Circadian or sleep disturbance, and misalignments between their phasing can lead to different types of diseases and disorders, most notably metabolic derangements.

It is clear that there is a rhythmic pattern in cell function and cycles of energy utilization in accordance with a daily rhythm 15, 16, while sleep plays a crucial role in maintaining metabolic homeostasis 17. However, the mechanism of bidirectional communication between the sleep centers and the circadian pacemaker, and their regulation of diverse metabolic networks is unclear (Fig. 1). In this article, we will outline the recent advances in our understanding of interdependencies amongst the circadian rhythms, sleep homeostasis, redox cycles, and other cellular energy metabolism networks. Potential applications of systems‐level investigations, applying integrated multi‐omics approaches to unravel cross‐talk between day/night cycles and metabolic systems will also be discussed.

## Beyond transcription/translation feedback loop (TTFL) mechanisms of the clockwork

Since the 1980s, transcriptional/translational feedback loops (TTFLs), wherein rhythmicity in the expression patterns of specific genes are controlled by the periodic expression of “clock” gene products, were considered as the principal drivers of circadian periodicity in multiple biological systems 1, 2, 18, 19. TTFL models indicate the presence of both positive and negative components in circadian clocks, where the positive loops activate transcription, while the negative elements inhibit the positive components in a cyclic manner. In the mammalian clock, BMAL1 and CLOCK proteins serve as the positive elements by forming a heterodimeric transcription factor complex that promotes expression of members of the *Period* (*Per*) and C*ryptochrome* (*Cry*) families 20. Subsequently, after entering the nucleus, the PER‐CRY heterodimers inhibit their own transcription by repressing the activity of the BMAL1‐CLOCK complex 21, 22. Eventually, a drop in the level of PERs and CRYs de‐represses BMAL1‐CLOCK activity to initiate a new cycle.

Information obtained through the TTFL‐based models is undoubtedly essential for understanding various aspects of circadian rhythms, and in particular tissue functions, since transcriptome and proteome alterations are extensive and cyclical in a range of studies 23, 24, 25, 26, 27, 28, 29, 30. However, in the past decade, identification of molecular rhythmicity in both prokaryotic and eukaryotic cells when transcription and translation are absent cannot be explained by existing models of TTFL mechanisms 31, 32, 33, 34. Moreover, previous observations based on analysis of clock gene mutants in both mammals and fruit flies require re‐interpretation in view of recent results demonstrating persistent circadian rhythms in these systems in which negative feedback within the TTFL loop is abolished 15, 35. Taken together, it is now apparent that TTFL‐based models for rhythmicity cannot provide a complete explanation for all features of circadian rhythmicity (reviewed in 36).

### Transcription‐translation feedback is not required for circadian oscillations in cyanobacteria

**Figure 2:**
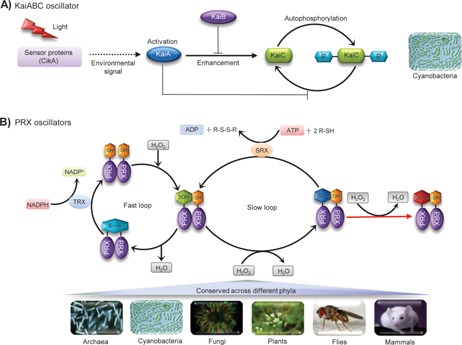
Molecular architecture of non‐transcriptional oscillators (NTOs) in prokaryotic and eukaryotic organisms. **A:** Autonomous oscillation in cyanobacterial KaiC phosphorylation. Environmental sensors such as CikA transfer signal cascades to initiate the interaction between KaiA and dephosphorylated KaiC hexamers, which subsequently stimulates autokinase activity (phosphorylation happens at multiple residues of KaiC). KaiC autokinase activity is inhibited by KaiB binding, which aids in maintaining the equilibrium state between non‐phosphorylated and phosphorylated forms of KaiC. **B:** Oxidation‐reduction cycles of the peroxiredoxin (PRX) proteins as a conserved biomarker of circadian clocks across distinct phyla. There are two interconnected cycles (fast and slow loop) in the catalytic mechanism of typical 2‐Cys PRXs. In the first cycle (fast loop) peroxidation of catalytic cysteine of PRXs leads to the formation of sulfenic acid (Cys‐SOH), followed by disulfide bond (S—S) formation. The recycling step is catalyzed by Thioredoxin (TRX). Further oxidation of sulfenic moiety of PRXs (Cys‐SOH) to sulfinic acid form (Cys‐SO_2_H) happens in the second cycle (slow loop). Overoxidized Cys‐SO_2_H residue can be slowly recycled through a reduction reaction carried out by sulfiredoxin (SRX) in an ATP‐dependent manner. The sulfinic form (Cys‐SO_2_H) can also be hyperoxidized into a sulfonic acid (Cys‐SO_3_H), but this transformation is thought to be irreversible (modified from 15).

Nearly 10 years ago, the major shortcomings of the TTFL‐based mechanism became obvious when circadian rhythms were found to be persisting in cyanobacteria even in the absence of transcription‐translation feedback 31. Identification of temperature‐compensated, self‐sustainable and robust oscillation in cyanobacterial KaiC phosphorylation evidently indicated transcription‐translation feedback could be important, but not indispensable for circadian rhythmicity 32. Intriguingly, subsequent studies provided valuable information regarding the dynamics of the circadian KaiABC oscillator and its modulation by redox‐active cofactor, which helped to decipher the precise mechanism of this entirely non‐transcriptional phosphorylation‐based rhythm in cyanobacteria 37, 38. Identification of the cyanobacterial KaiABC oscillator indicated the existence of non‐transcriptional oscillators (NTOs); nevertheless, the analysis of such an NTO was only restricted to cyanobacteria, as Kai proteins are not conserved across distinct phyla (Fig. 2A).

### Peroxiredoxins serve as the conserved biomarkers of circadian clocks

Another peculiarity about circadian systems previously was the apparent lack of molecular phylogeny of mechanisms controlling circadian rhythms. Virtually all known “clock” genes and proteins are not conserved across the various domains of life, although there are some homologous components in fruit flies and mammals. This previously suggested that the “logic” of the clockwork always involved a TTFL, but the players were different in each model system that has been studied 39. Of late, the oxidation‐reduction status of peroxiredoxin (PRX) proteins has been found to be regulated in a rhythmic fashion in anucleate human red blood cells (RBCs) without involving any transcriptional‐translational mechanisms, representing the presence of autonomous oscillations in the redox status of the cell 33, 34. Subsequently, oscillations of peroxiredoxin proteins (PRX) have been established as evolutionarily conserved markers of the clockwork, pointing to redox cycles as a likely unifying principle among disparate organisms 40, which was not observed in case of KaiABC oscillators (Fig. 2B).

PRXs are thiol‐dependent peroxidases, which serve as an antioxidant in the maintenance of intracellular levels of hydrogen peroxide (H_2_O_2_), peroxinitrite, and hydroperoxides to protect organisms against diverse oxidative stresses. Interestingly, these abundant cellular antioxidant proteins, which are probably evolved from a thioredoxin‐like ancestor 41, are present in almost all living organisms 42. On the basis of the number and bonding pattern of the catalytic cysteine residues, PRXs are classified in two distinct groups. The 1‐Cys family contains only the NH_2_‐terminal Cys that can become oxidised, while the 2‐Cys type contains both the NH_2_‐ and COOH‐terminal Cys residues and can therefore form either *inter*molecular (typical 2‐Cys Prx) or *intra*molecular (atypical 2‐Cys Prx) disulfide bonds during oxidation. Apart from the detoxification of the various peroxide substrates, eukaryotic 2‐Cys peroxiredoxins (2‐Cys PRXs) play a significant role in hydrogen peroxide‐mediated signal transduction pathways 43. In addition to cyclic oxidation of peroxiredoxin proteins, it is highly likely that redox oscillations impact directly on many other susceptible proteins in cells. Specifically, so‐called hyper‐reactive cysteine residues represent particularly attractive targets 44.

### Do the non‐transcriptional and TTFL oscillators and redox state regulate each other?

Redox state may impact on transcriptional activity of clock components 45 and neuronal activity within the master pacemaker in mammals, the suprachiasmatic nuclei (SCN) 46. Interestingly, in turn circadian clocks also control the cellular redox status, since expression levels of many reactive oxygen species (ROS) responsive genes, or antioxidant enzymes, are frequently regulated by the clock genes 47, 48. Consequently, mechanistic interactions between redox and circadian components suggest that the redox state of a cell and clocks are influenced and regulated by each other.

The existence of NTOs such as the cyanobacterial KaiABC oscillator, and the more pervasive oxidation‐reduction cycles of PRX proteins, thus challenges the paradigm of TTFL‐based mechanisms. Now, the imperative question is whether the NTO and TTFL‐based oscillators are interlinked, and if so, how they co‐exist in cells and collectively interplay to maintain unequivocal cellular time keeping. Although direct connections between the two systems is not yet fully delineated, there is experimental evidence for their interactions, and mathematical modeling indicates that coupling between NTO and TTFLs effectively boosts overall clock performance (reviewed in 36, 49). First of all, the Kai and PRX system still exhibit oscillation even in the absence of TTFL 32, 33. Secondly, circadian rhythms are not obliterated in systems with either constitutive expression or deletion of clock genes (in TTFL knockout mutants) 35, 50, 51. It is therefore reasonable to speculate that timekeeping is controlled by a biochemical oscillator. To this end, understanding of the reciprocal communication of circadian oscillators with various metabolic and redox cycles could provide valuable insights, as elaborated in the next section.

## Cross‐talk between the circadian and metabolic clocks: Reciprocal regulation of the circadian cycles and energetic pathways

Connection between circadian and metabolic systems is one of the most important and enlightening areas of current circadian biology research 52, 53, 54, 55. Adverse effects of circadian disruption or sleep deprivation on metabolic functions clearly indicate the impact of these processes on energy homeostasis 56. Over a decade ago, McKnight and co‐workers demonstrated regulation of two clock proteins (BMAL1 and CLOCK) by the metabolic cofactor NAD(P), and therefore, a connection between the cellular metabolism and clockwork circuitry 57.

**Figure 3:**
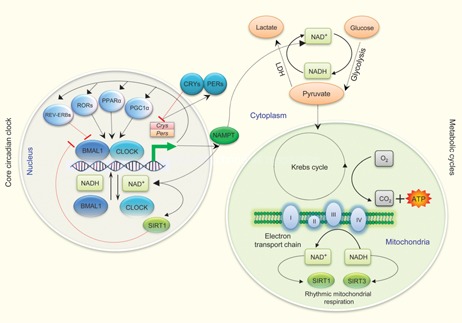
Cross‐talk between the circadian and metabolic clocks. Coupling mechanisms between the circadian and metabolic oscillators are miscellaneous. There are a series of transcription/translation feedback loops in the core clock mechanism. CRY proteins (along with the PER proteins) function as the negative regulators for maintenance of circadian rhythms. PPARα and PGC‐1α stimulates expressions of clock genes, while RORs regulate Bmal1 transcription through formation of a feedback loop involving RORα and REV‐ERBα. Core clock proteins such as BMAL1 and CLOCK (NPAS2 substitutes for CLOCK in some brain regions (not shown)) regulate the rate limiting steps of NAD+ biosynthesis 58, 62, while the DNA binding affinity of BMAL1 and CLOCK is controlled by the intracellular NAD+/NADH ratio 45. LDH plays a crucial role in increasing the cellular concentration of NAD+. NAD+‐dependent deacetylases, for example SIRT1 or SIRT3 regulate circadian clock gene expression 59, 64. NAMPT acts as a rate‐limiting enzyme in mammalian NAD+ biosynthesis and its expression is also regulated by the core clock genes 62. (Details for these possible connecting components between the circadian oscillators and various metabolic processes have been summarised in Table 1). Abbreviations: BMAL1, brain and muscle ARNT‐Like 1; CLOCK, circadian locomotor output cycles kaput; Cry, cryptochrome; LDH, lactate dehydrogenase; NAD, nicotinamide adenine dinucleotide; NAMPT, nicotinamide phosphoribosyl‐transferase; NPAS2, neuronal PAS domain protein 2; Per, period; PPAR, peroxisome proliferators‐activated receptor; PGC‐1α, PPAR gamma coactivator‐1 alpha; ROR, retinoic acid orphan receptors; SIRT 1, sirtuin 1; SIRT 3, sirtuin 3.

With the passage of time, subsequent studies have illustrated several modes of bidirectional regulation of cellular metabolites and clock proteins. For example, there is modulation of CLOCK‐mediated chromatin remodeling and regulation of circadian clock gene expression by the NAD+‐dependent deacetylase SIRT1 58, 59 and circadian regulation of the NAD+ salvage pathway by CLOCK‐SIRT1 60. Similarly, circadian regulation of the enzymatic activity of acetyl‐CoA Synthetase 1 (AceCS1) leads to varying intracellular levels of the central metabolite acetyl‐CoA 61. Furthermore, nicotinamide phosphoribosyltransferase (NAMPT), the rate‐limiting enzyme in mammalian NAD+ biosynthesis, exhibits rhythmicity in its expression level 62. These observations highlight how the clock's downstream transcriptional network extends to metabolic genes. Likewise, other metabolic links have been elaborated, in particular with respect to poly(ADP‐ribose) polymerase 1 (PARP‐1) 63 and the role of the NAD(+)‐dependent deacetylase sirtuin 3 (SIRT3) in rhythmic mitochondrial function 64 (Fig. 3). Connecting hubs between the metabolic and clock networks are diverse, and certainly not yet fully explored. However, in the light of our current understanding of circadian biology, it is likely that NAD‐dependent enzymes, nutrient‐sensing transcriptional regulatory proteins, redox transcription factors, and protein kinases serve as the crucial candidates for mediating cross‐talk between energetic pathways and circadian cycles 65. Table 1 summarizes the circadian control of different metabolic processes in eukaryotes.

**Table 1:** Possible connecting components between the circadian oscillators and metabolic processes

Candidates	Plausible roles/ involvement in cross‐talks	References
CLOCK/NPAS2	Circadian transcription factor CLOCK/NPAS2 controls NAD+ biosynthesis through regulation of NAMPT expression	62
	NAD(P)+/NAD(P)H ratio regulates the binding of CLOCK/NPAS2‐BMAL1 heterodimers to their E box cognate sequence	
PER proteins	PER proteins regulate expression of the core clock gene Bmal1	59, 115
	Binding of SIRT1 to CLOCK‐BMAL1 complexes promotes PER2 deacetylation and degradation	
	PER proteins regulates lipid and glycogen metabolism through their interactions with diverse nuclear receptors	
CRY proteins	CRY proteins (along with the PER proteins) function as the negative regulators for maintenance of circadian rhythms	69, 116
	They regulate circadian rhythmicity of cAMP signaling and hepatic gluconeogenesis	
	AMPK directly phosphorylates CRY proteins and reduces their half‐life	
NAD^+^	NAD(P)^+^ and NAD(P)H reflect the metabolic and redox status of the cell	58, 62
	NAD^+^ serves as a metabolic oscillator and controls the core clock machinery primly through SIRT1	
SIRT1	NAD^+^‐dependent SIRT1 controls expression the circadian clock genes (Bmal1, Per2, and Cry1) through PER2 deacetylation	58, 59, 60
	CLOCK‐SIRT1 regulates circadian control of the NAD+ salvage pathway	
	It regulates circadian transcription also by the deacetylation of histone H3 tails	
SIRT3	SIRT3 maintains rhythms in the acetylation and activity of oxidative enzymes and respiration	64
	Core clock components regulate its activity through control of concentrations of NAD^+^	
NAMPT	NAMPT is the rate‐limiting enzyme in mammalian NAD+ biosynthesis	62
	Its expression is regulated by the core clock genes	
	Its inhibition leads to the oscillation of Per2 by releasing CLOCK: BMAL1	
PARP1	PARP1 modifies clock components in response to feeding‐fasting cycles	63, 117
	It regulates the binding of CLOCK‐BMAL1 to DNA and interaction of CLOCK‐BMAL1 with PER and CRY repressor proteins	
	SP1, a nuclear target protein of PARP‐1, regulate its expression	
PRXs	PRX proteins exhibit self‐sustained oscillation in their oxidation–reduction cycles	34, 40
	PRX cycle provides feedback to regulate the core clock transcriptional network probably through the oscillation of ROS	
	Perturbation of their functions causes a long‐period phenotype or leads to a depression in the amplitude of circadian oscillations	
AceCS1	Circadian control of intracellular levels of acetyl‐CoA and thereby fatty acid elongation is regulated through the enzymatic activity of AceCS1	118
	AceCS1 activity is controlled by acetylation, and its rhythmic acetylation is regulated by SIRT1	
AMPK	AMPK serves as the major sensor of the AMP/ATP ratio, activates stress‐promoted transcription and regulates clock gene expression	119
	It regulates stability (promotes degradation) of core clock proteins (CRY and PER)	
PPARα and PGC‐1α	Transcriptional coactivator PGC‐1α stimulates expressions of clock genes (Bmal1 and Rev‐erbα)	120, 121
	It has association with the SirT1 histone deacetylase complex, can serve as a sensor for the metabolic state of the cell, and also induces the expression of gluconeogenic genes	
	PPARα regulates fatty acid oxidation and apolipoprotein synthesis	
ALAS1	ALAS1, the rate limiting enzyme in haem biosynthesis, is a target gene for the NPAS2/BMAL1 heterodimer	122
	Circadian rhythmicity in the cellular haem levels in maintained through the regulation of the expression of ALAS1 by the core clock genes	
	Reciprocally, haem regulates activity of the BMAL1‐NPAS2 transcription complex	
HSF1	HSF1 plays an important role in transporting nutrient signals to the circadian circuitry	123
	Phosphorylation by diverse protein kinases regulates its activity	
	It also functions as a key regulator of temperature‐dependent expression of heat shock protein/ chaperone genes associated with circadian oscillators	
CREB	cAMP signaling via CREB and other transcriptional oscillator is imperative for the molecular circadian oscillators	124, 125
	CREB‐dependent transcription supports steady cycling of the core clock transcriptional loop	
FOXO	Nutrient and stress sensor FOXO regulates sensitivity of the circadian clock to stress conditions; its effects on circadian rhythms are non‐cell‐autonomous	126, 127
	SIRT1 regulates FOXO transcription factors in a stress‐dependent manner	
	Expression of several gluconeogenic genes is directly regulated by FOXO1	
RORs	RORs are components of the master oscillator in mammalian circadian system that regulate Bmal1 transcription through formation of a feedback loop involving RORα and REV‐ERBα	128, 129
	RORs can alter PER2 activity by direct physical interactions	

AceCS1, Acetyl‐CoA Synthetase 1; ALAS1, aminolevulinate synthase 1; AMPK, AMP‐dependent protein kinase; CREB, cAMP response element‐binding protein; Cry, cryptochrome; FOXO, Forkhead homeobox type O; HSF1, heat shock transcription factor 1; NAD, nicotinamide adenine dinucleotide; NAMPT, nicotinamide phosphoribosyl‐transferase; NPAS2, neuronal PAS domain protein 2; PARP1, poly (ADP‐ribose) polymerase 1; Per, period; PPAR, peroxisome proliferators–activated receptor; PGC‐1α, PPAR gamma coactivator‐1 alpha; PRX, peroxiredoxin; ROR, retinoic acid orphan receptors; ROS, reactive oxygen species; SIRT, sirtuin.

Oscillation of metabolic pathways is not a new concept. In the 1960s, rapid rhythmicity (over minutes rather than hours) in the glycolytic intermediates glucose‐6‐phosphate/fructose‐6‐phosphate and fructose‐1,6‐diphosphate (FDP) levels was first demonstrated in yeast extracts by Ghosh and Chance 66, and more recent studies have built on this initial work 67, 68. On a longer timescale, hepatic gluconeogenesis, which plays an important role in maintaining glucose homeostasis in mammals during starvation, is also under circadian regulation, most probably through the control of Cryptochromes (*Cry1* and *Cry2*) by CLOCK and BMAL1 69. Similarly, components of the Krebs cycle such as NADP‐dependent isocitrate dehydrogenase also exhibit circadian periodicity in their abundance or activity 70.

Intriguingly, several components of central metabolic pathways, such as a number of rate‐limiting enzymes involved in glycolysis and the tricarboxylic acid (TCA) cycle, are redox‐sensitive, indicating the potential of these metabolic pathways to be regulated by the circadian redox oscillations 71. A key metabolite at the interface of cytosolic and mitochondrial metabolism is acetyl‐CoA, which not only plays a role in metabolism itself, but also regulates protein function by participating in acetylation reactions. Such post‐translational modification of proteins, which include histones, can thus regulate gene expression, in addition to modulating enzyme function. In this vein, recent work highlights that clock‐driven acetylation modulates a considerable number of mitochondrial proteins involved in multiple metabolic networks 72. Moving beyond the analysis of transcriptional processes and gene expression patterns is essential to address connectivity between the clock and metabolism, but has been challenging because of the technical challenges posed by performing such analyses, which rely heavily on mass spectrometry based metabolomics.

Recently, comprehensive metabolomics analyses of different biological fluids (saliva and blood) have provided insights regarding the circadian regulation of various human metabolic pathways 73, and consequences of sleep deprivation on the human metabolome 74. Of note, it has been demonstrated that nearly 15% of all metabolites identified in human plasma and saliva are controlled by circadian clocks 73. Similarly, a good number of metabolites in exhaled human breath also exhibit circadian rhythmicity 75. Importantly, sleep deprivation in humans adversely affects the oscillatory behavior of many blood metabolites including tryptophan, serotonin, taurine, acylcarnitines, glycerophospholipids, and sphingolipids 74, indicating a connection between sleep restriction and circadian clock disruption and metabolic dysfunction. Further work is, however, needed to delineate the exact mechanism for these observations. These studies have thus been important in creating an avenue towards the recognition of new physiological/metabolic pathways which are controlled by circadian clocks or sleep‐wake cycles. Moreover, such metabolomic profiling has the potential to identify novel noninvasive biomarkers of circadian disruption, sleep deprivation and associated metabolic and neurological disorders.

## How might the clock and sleep be connected?

In mammalian circadian system, the brain's suprachiasmatic nucleus (SCN), which is considered as the master circadian “clock,” orchestrates synchronization of oscillators in peripheral tissues 76, 77. The neural circuits involved in the regulation of sleep‐wake states and circadian rhythms are becoming established, as are the vital roles of circadian and homeostatic processes in regulation of the sleep and arousal‐promoting circuitry 7, 78. Intriguingly, there is physiological evidence that indicate sleep centers can also regulate the circadian pacemaker 79. In addition, sleep plays a role in the clearance of potentially neurotoxic waste products from the central nervous system, and thereby maintains metabolic homeostasis 17. Circadian clocks regulate different aspects of sleep, suggesting that redox and metabolism may affect sleep homeostasis through their impact on the state of the circadian system. However, the exact mechanism by which sleep‐wake centers communicate with the SCN and metabolic cycles has not been untangled.

Components of the central circadian pacemaker are known to regulate sleep onset and control the transition to wakefulness, while the quantity of sleep appears to be controlled by homeostatic centers 80. At the molecular level, the interactions between clocks and sleep‐wake cycles have been investigated using mutant mice and fruit fly models lacking core circadian clock genes to try to unravel the possible functions of clock genes in sleep homeostasis (reviewed in 81, 82). For example, targeted disruption or deletion of the core clock components such as *Bmal1* (*Cycle* in *Drosophila*), *Clock/Npas2*, *Per1/Per2*, and *Cry1*/*Cry2* result in various phenotypes including increased sleep fragmentation and rebound following deprivation of sleep, in addition to an enhanced tendency to switch between non‐rapid eye movement (NREM) and rapid eye movement (REM) sleep 83. Conversely, sleep deprivation can impinge on the expression levels of core circadian transcriptional regulators and their DNA‐binding capabilities 84, demonstrating the existence of a reciprocal regulation between the central clock machinery and sleep‐wake cycle. Interestingly, as discussed in the previous section, binding of certain clock proteins may be regulated by intracellular redox potential, indicating the potential for cross‐talk between the circadian clock machinery, energy metabolism, and sleep regulation.

There is a longstanding belief that sleep happens only at the level of the whole organism. However, recent work highlights that even within the brain, there is local and use‐dependent sleep of subsets of neural circuitry, which forces a reappraisal of what sleep is and what is might be for 85, 86. For example, electrophysiological analysis of the sleep‐wake cycle indicates the concurrent existence of different sleep intensities within distinct regions of human brain 87. Likewise, slow wave activity (SWA) in local cortical EEG recordings from brain regions of awake animals demonstrates that neuronal subsets may enter “off” states during a long stretch of wakefulness, probably due to the falling in levels of arousal‐promoting neuromodulators 88. Similarly, Krueger and colleagues have demonstrated sleep as a property of local neuronal assemblies, and hypothesized that the local‐network sleeps, which are perhaps controlled by the oscillation in the levels of sleep‐regulatory molecules, are connected by central mechanisms and serve as the fundamental basis of whole‐organism sleep 89. Therefore, local populations of neurons might sleep at the cellular level in a use‐dependent manner, which could be a means of saving energy, particularly during prolonged wakefulness. To this end, it would be interesting to decipher the possible functional consequences of such local characteristics of sleep.

Akin to circadian clocks, it is now imperative to study the self‐sustained mechanism of sleep at the cellular level, which is often difficult to execute in vivo in the mammalian brain due to the presence of the entangled web of neuronal networks that are controlled by signals from both local and global sources. Of note, a recent study on primary mouse cortical cultures demonstrated sleep as a prominent characteristics of simple neuronal networks grown in vitro 90. Interestingly, the authors observed electrophysiological, metabolic and transcriptomic similarities between the in vitro neuronal networks and those determined from sleep‐deprived mice. The presence of important features of the sleep‐wake cycle in an in vitro setting opens up new avenues for molecular level investigations of the local nature of sleep and its multifaceted interplay with diverse metabolic and circadian oscillators, which has not been possible to achieve previously.

## The promise of systems level multi‐omics approaches to unraveling the interconnectivity of circadian clockworks, metabolic oscillators, and the sleep‐wake switch

Although identification of NTOs and metabolic oscillations has enhanced our understanding of circadian rhythmicity, there are still several opaque areas that entail further investigation (Fig. 1). The most contemporary questions are: (1) In what manner do non‐transcriptional and transcriptional clock mechanisms interact with each other? (2) What are the mechanisms underlying the reciprocal regulation of the non‐transcriptional circadian clocks and energetic pathways? (3) Do circadian redox oscillations impact on the sleep‐wake switch? (4) How are dysfunctions in circadian clocks/sleep‐wake cycles linked to diverse diseases, including metabolic disorders such as diabetes mellitus?

### Proteome level analyses can serve as an excellent complementary platform for mRNA level observations

**Figure 4:**
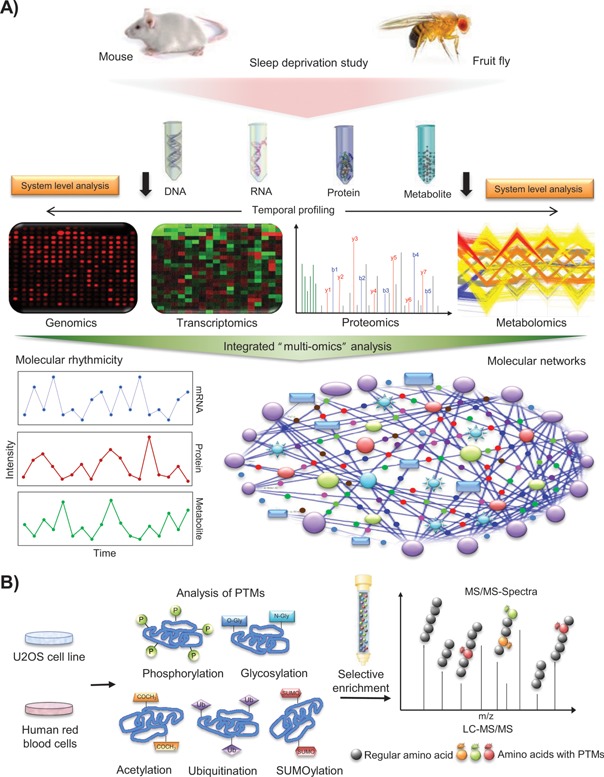
Systems level multi‐omics approaches to uncover the bi‐directional communications between the sleep centers and the circadian pacemaker. **A:** Schematic representation of an integrated multi‐omics (genomics, transcriptomics, proteomics, and metabolomics) analysis for unraveling the interconnectivities among circadian clocks, metabolic cycles and the sleep‐wake switch. Systems level analyses of sleep‐deprived models (fruit flies and mice) could collectively provide an inclusive representation regarding the temporal physiological states of organisms, and alterations in molecular oscillations and networks as a consequence of sleep deficiency. **B:** Schematic illustration of an analysis of post‐translational modifications (PTMs) in a non‐transcriptional (anucleate red blood cell) model and a nucleated cell line (human osteosarcoma U2OS cell line) in vitro for studying the connectivity between the non‐transcriptional and TTFL mechanisms of circadian rhythmicity. Selective enrichment of the post‐translationally modified peptides and subsequent mass spectrometry‐based profiling of diverse PTM patterns might provide additional mechanistic insights into the dynamic modifications of core clock proteins and their interactors.

While transcriptomic studies have previously provided valuable insights into which gene networks and tissue‐specific programmes are controlled by the circadian clock 23 and sleep 5, 91, there has been a dearth of proteome level investigation in circadian biology 92. As with metabolomics analyses, technological limitations have previously hampered progress in global protein profiling. Initial proteomics studies implemented gel‐based approaches, and consequently, were only able to measure expression patterns of a limited number of rhythmic proteins due to the poor coverage of the entire proteomes 28, 93, 94. Application of next‐generation quantitative proteomics approaches involving ultra‐sensitive mass spectrometers, which are presently at the pinnacle of promising proteomics technologies 95, could be extremely useful in decoding the mechanisms and extent of protein oscillations and the links to the sleep‐wake switch (Fig. 4A). With the broadening availability of mass spectrometry, a number of quantitative proteomics datasets have emerged investigating the mechanism of synchronization of circadian rhythms by the SCN 96, 97, post‐transcriptional mechanisms of circadian regulation 30, and diurnal oscillations in the mammalian hepatic proteome 29. To date, such approaches have not been applied to characterize sleep‐wake cycles, or to determine proteome level changes upon sleep deprivation, although gel‐based techniques have been employed to characterize the latter in the past 98, 99.

In recent years, ribosome profiling, which provides genome‐wide information on protein synthesis through deep sequencing of ribosome protected mRNA fragments, is emerging as an efficient technique to track in vivo translation. Circadian clocks play some vital roles in coordinating transcription and translation steps which are essential for ribosome biogenesis. Intriguingly, some very recent studies have demonstrated the promising applications of ribosome profiling for studying translational control in circadian gene expression and for mapping rhythmic translatome 100, 101. Proteomic measurements and ribosome profiling collectively can provide comprehensive information regarding rhythmic proteins and can complement the technical limitations of each others.

### Redox proteomics and characterization of post‐translational modifications provide novel mechanistic insights into circadian biology

Cross‐talk between the cellular redox state and the circadian clocks has been studied extensively in different organisms 45, 102, 103. Following the identification of oxidation‐reduction cycles of peroxiredoxin proteins as the conserved markers of circadian rhythms 40, there has been an emerging interest in profiling redox oscillations at a global level to investigate the consequences of metabolic cycles on circadian rhythmicity and sleep‐wake switching. Recent studies indicate that the circadian rhythm of redox state controls excitability in SCN neurons 46, 104. Consequently, it can be speculated that redox homeostasis and neuronal activity are coupled non‐transcriptional circadian oscillators intertwined in neuronal physiology. Peroxiredoxin proteins may not be unique in their ability to undergo redox oscillations since many other proteins are susceptible to oxidation of their cysteine residues by peroxide 44. Thus, redox proteomics analyses will undoubtedly provide a novel mechanistic insight into the effects of brain disruption of redox processes/signaling on sleep and circadian rhythms and vice versa 71.

A complementary set of analyses could also be used to probe specific post‐translational modifications (PTMs) of proteins across the day, and also under sleep restriction paradigms. Such modifications regulate the recruitment, recognition, assembly/disassembly, translocation, and consequently the eventual fates of the majority of eukaryotic proteins 105. In the context of the clockwork, there is substantial evidence that the control of functional activity and stability of well characterized clock proteins is driven by diverse types of PTMs, including phosphorylation 106, acetylation 58, 59, ubiquitylation 107, and SUMOylation 108. We thus anticipate that comparative analysis of PTMs in non‐transcriptional models (e.g. anucleate red blood cells) and nucleated cell culture lines (e.g. human osteosarcoma U2OS cell line) have the potential to identify dynamic modifications in proteins that might be known to be associated with the clock, but also others that are completely novel. Moreover, examining non‐transcriptional and transcriptional models will establish nodes of interconnectivity between these mechanisms (Fig. 4B).

### Integration of different “omics” datasets is promising for studying functional interactions between circadian and metabolic cycles

A recent study by Sassone‐Corsi and colleagues demonstrated synergistic links between oscillations in the circadian transcriptome and metabolic pathways 109. Furthermore, the same research group has developed the CircadiOmics platform (http://circadiomics.igb.uci.edu/), which is a computational framework that could serve as a repository for metabolomics and other “omics” level high‐throughput data associated with circadian clocks 110. Nevertheless, existing tools such as this do not yet integrate proteome level information, which is critical for building a comprehensive view of biological rhythmicity. Thus far, similar integrative endeavors have not been attempted for sleep datasets, and therefore tying together common molecular pathways between sleep and the circadian clock at a systems level remains an unrealized goal.

In order to unravel the transient snapshots of dynamic circadian rhythmicity and sleep homeostasis, there is an urgent need to amalgamate the findings obtained from mRNA, protein and metabolite level investigations to get a true understanding of these multiple dynamic facets. In the future, we anticipate that an integrated multi‐omics analysis (specifically proteomics and metabolomics) of sleep‐deprived models (using different complementary model organisms such as the mouse and fruit fly) will collectively provide a complete representation of bidirectional molecular communication between the sleep centers and the circadian pacemaker. Eventually, this may also lead to the identification of novel conserved markers of circadian rhythms and sleep deprivation (Fig. 4A). Consequently, integrated quantitative multi‐omics analyses may also highlight molecular pathways affected by disruption of circadian clocks and sleep‐wake cycles and their association with different adverse health consequences.

## Conclusions and outlook

We now have a vast array of knowledge about the molecular underpinnings of the circadian clock, particularly at the level of transcriptional networks. The influence of the clock is pervasive, with ∼40% of transcripts in the mouse genome displaying daily cycles 111. Similarly, transcriptional changes in the brain under sleep deprivation are extensive. However, how sleep and the clock are linked at the molecular level remains a fascinating question. Novel insights into metabolic cycles and their connectivity with the circadian clockwork promise to offer potential routes to link the two processes, since sleep is also influenced by metabolism 112, 113 and it regulates metabolic processes within the body 114. A considerable amount of “dark matter” still, however, exists in our understanding of the clockwork and sleep, which stems from a lack of tools to assess other “omes” at a global, quantitative level. In particular, we vitally need proteomic datasets that not only quantify protein abundance, but also a diversity of post‐translational modifications that can modulate their function. Moreover, we must concurrently assay metabolites to get a complete picture of the end products of metabolic pathways that are known to be regulated by the circadian and sleep systems. Multi‐omics approaches that are now becoming widely accessible will thus change the way that high‐throughput temporal profiling can be performed, and thus offers a conduit to insights that have previously been beyond our reach.

## References

[1] Dunlap JC . 1999 Molecular bases for circadian clocks. Cell 96: 271–90. 9988221 10.1016/s0092-8674(00)80566-8

[2] Bell‐Pedersen D , Cassone VM , Earnest DJ , Golden SS , et al. 2005 Circadian rhythms from multiple oscillators: lessons from diverse organisms. Nat Rev Genet 6: 544–56. 15951747 10.1038/nrg1633 PMC2735866

[3] Siegel JM . 2005 Clues to the functions of mammalian sleep. Nature 437: 1264–71. 16251951 10.1038/nature04285 PMC8760626

[4] Nitz DA , van SB , Tononi G , Greenspan RJ . 2002 Electrophysiological correlates of rest and activity in *Drosophila melanogaster* . Curr Biol 12: 1934–40. 12445387 10.1016/s0960-9822(02)01300-3

[5] Maret S , Dorsaz S , Gurcel L , Pradervand S , et al. 2007 Homer1a is a core brain molecular correlate of sleep loss. Proc Natl Acad Sci USA 104: 20090–5. 18077435 10.1073/pnas.0710131104 PMC2148427

[6] Broussard JL , Ehrmann DA , Van CE , Tasali E , et al. 2012 Impaired insulin signaling in human adipocytes after experimental sleep restriction: a randomized, crossover study. Ann Intern Med 157: 549–57. 23070488 10.7326/0003-4819-157-8-201210160-00005 PMC4435718

[7] Saper CB , Scammell TE , Lu J . 2005 Hypothalamic regulation of sleep and circadian rhythms. Nature 437: 1257–63. 16251950 10.1038/nature04284

[8] Schwartz JR , Roth T . 2006 Shift work sleep disorder: burden of illness and approaches to management. Drugs 66: 2357–70. 17181377 10.2165/00003495-200666180-00007

[9] Reddy AB , O'Neill JS . 2010 Healthy clocks, healthy body, healthy mind. Trends Cell Biol 20: 36–44. 19926479 10.1016/j.tcb.2009.10.005 PMC2808409

[10] Durgan DJ , Young ME . 2010 The cardiomyocyte circadian clock: emerging roles in health and disease. Circ Res 106: 647–58. 20203314 10.1161/CIRCRESAHA.109.209957 PMC3223121

[11] Vetter C , Fischer D , Matera JL , Roenneberg T . 2015 Aligning work and circadian time in shift workers improves sleep and reduces circadian disruption. Curr Biol 25: 907–11. 25772446 10.1016/j.cub.2015.01.064

[12] Hastings MH , Reddy AB , Maywood ES . 2003 A clockwork web: circadian timing in brain and periphery, in health and disease. Nat Rev Neurosci 4: 649–61. 12894240 10.1038/nrn1177

[13] Wulff K , Gatti S , Wettstein JG , Foster RG . 2010 Sleep and circadian rhythm disruption in psychiatric and neurodegenerative disease. Nat Rev Neurosci 11: 589–99. 20631712 10.1038/nrn2868

[14] Sahar S , Sassone‐Corsi P . 2009 Metabolism and cancer: the circadian clock connection. Nat Rev Cancer 9: 886–96. 19935677 10.1038/nrc2747

[15] Reddy AB , Rey G . 2014 Metabolic and non‐transcriptional circadian clocks: eukaryotes. Annu Rev Biochem 83: 165–89. 24606143 10.1146/annurev-biochem-060713-035623 PMC4768355

[16] Rey G , Reddy AB . 2013 Physiology. Rhythmic respiration. Science 342: 570–1. 24179212 10.1126/science.1246658 PMC4768352

[17] Xie L , Kang H , Xu Q , Chen MJ , et al. 2013 Sleep drives metabolite clearance from the adult brain. Science 342: 373–7. 24136970 10.1126/science.1241224 PMC3880190

[18] Takahashi JS , Hong HK , Ko CH , McDearmon EL . 2008 The genetics of mammalian circadian order and disorder: implications for physiology and disease. Nat Rev Genet 9: 764–75. 18802415 10.1038/nrg2430 PMC3758473

[19] Harmer SL , Panda S , Kay SA . 2001 Molecular bases of circadian rhythms. Annu Rev Cell Dev Biol 17: 215–53. 11687489 10.1146/annurev.cellbio.17.1.215

[20] Chen R , Schirmer A , Lee Y , Lee H , et al. 2009 Rhythmic PER abundance defines a critical nodal point for negative feedback within the circadian clock mechanism. Mol Cell 36: 417–30. 19917250 10.1016/j.molcel.2009.10.012 PMC3625733

[21] Sangoram AM , Saez L , Antoch MP , Gekakis N , et al. 1998 Mammalian circadian autoregulatory loop: a timeless ortholog and mPer1 interact and negatively regulate CLOCK‐BMAL1‐induced transcription. Neuron 21: 1101–13. 9856465 10.1016/s0896-6273(00)80627-3

[22] Kume K , Zylka MJ , Sriram S , Shearman LP , et al. 1999 MCRY1 and mCRY2 are essential components of the negative limb of the circadian clock feedback loop. Cell 98: 193–205. 10428031 10.1016/s0092-8674(00)81014-4

[23] Akhtar RA , Reddy AB , Maywood ES , Clayton JD , et al. 2002 Circadian cycling of the mouse liver transcriptome, as revealed by cDNA microarray, is driven by the suprachiasmatic nucleus. Curr Biol 12: 540–50. 11937022 10.1016/s0960-9822(02)00759-5

[24] Panda S , Antoch MP , Miller BH , Su AI , et al. 2002 Coordinated transcription of key pathways in the mouse by the circadian clock. Cell 109: 307–20. 12015981 10.1016/s0092-8674(02)00722-5

[25] Storch KF , Lipan O , Leykin I , Viswanathan N , et al. 2002 Extensive and divergent circadian gene expression in liver and heart. Nature 417: 78–83. 11967526 10.1038/nature744

[26] Ueda HR , Chen W , Adachi A , Wakamatsu H , et al. 2002 A transcription factor response element for gene expression during circadian night. Nature 418: 534–9. 12152080 10.1038/nature00906

[27] Ueda HR , Hayashi S , Chen W , Sano M , et al. 2005 System‐level identification of transcriptional circuits underlying mammalian circadian clocks. Nat Genet 37: 187–92. 15665827 10.1038/ng1504

[28] Reddy AB , Karp NA , Maywood ES , Sage EA , et al. 2006 Circadian orchestration of the hepatic proteome. Curr Biol 16: 1107–15. 16753565 10.1016/j.cub.2006.04.026

[29] Mauvoisin D , Wang J , Jouffe C , Martin E , et al. 2014 Circadian clock‐dependent and ‐independent rhythmic proteomes implement distinct diurnal functions in mouse liver. Proc Natl Acad Sci USA 111: 167–72. 24344304 10.1073/pnas.1314066111 PMC3890886

[30] Robles MS , Cox J , Mann M . 2014 In‐vivo quantitative proteomics reveals a key contribution of post‐transcriptional mechanisms to the circadian regulation of liver metabolism. PLoS Genet 10: e1004047. 24391516 10.1371/journal.pgen.1004047 PMC3879213

[31] Nakajima M , Imai K , Ito H , Nishiwaki T , et al. 2005 Reconstitution of circadian oscillation of cyanobacterial KaiC phosphorylation in vitro. Science 308: 414–5. 15831759 10.1126/science.1108451

[32] Tomita J , Nakajima M , Kondo T , Iwasaki H . 2005 No transcription‐translation feedback in circadian rhythm of KaiC phosphorylation. Science 307: 251–4. 15550625 10.1126/science.1102540

[33] O'Neill JS , van OG , Dixon LE , Troein C , et al. 2011 Circadian rhythms persist without transcription in a eukaryote. Nature 469: 554–8. 21270895 10.1038/nature09654 PMC3040569

[34] O'Neill JS , Reddy AB . 2011 Circadian clocks in human red blood cells. Nature 469: 498–503. 21270888 10.1038/nature09702 PMC3040566

[35] Lakin‐Thomas PL . 2006 Transcriptional feedback oscillators: maybe, maybe not. J Biol Rhythms 21: 83–92. 16603673 10.1177/0748730405286102

[36] van OG , Millar AJ . 2012 Non‐transcriptional oscillators in circadian timekeeping. Trends Biochem 37: 484–92. 10.1016/j.tibs.2012.07.006 22917814

[37] Wood TL , Bridwell‐Rabb J , Kim YI , Gao T , et al. 2010 The KaiA protein of the cyanobacterial circadian oscillator is modulated by a redox‐active cofactor. Proc Natl Acad Sci USA 107: 5804–9. 20231482 10.1073/pnas.0910141107 PMC2851934

[38] Qin X , Byrne M , Mori T , Zou P , et al. 2010 Intermolecular associations determine the dynamics of the circadian KaiABC oscillator. Proc Natl Acad Sci USA 107: 14805–10. 20679240 10.1073/pnas.1002119107 PMC2930409

[39] Rosbash M . 2009 The implications of multiple circadian clock origins. PLoS Biol 7: e62. 19296723 10.1371/journal.pbio.1000062 PMC2656552

[40] Edgar RS , Green EW , Zhao Y , van OG , et al. 2012 Peroxiredoxins are conserved markers of circadian rhythms. Nature 485: 459–64. 22622569 10.1038/nature11088 PMC3398137

[41] Copley SD , Novak WR , Babbitt PC . 2004 Divergence of function in the thioredoxin fold suprafamily: evidence for evolution of peroxiredoxins from a thioredoxin‐like ancestor. Biochemistry 43: 13981–95. 15518547 10.1021/bi048947r

[42] Hall A , Karplus PA , Poole LB . 2009 Typical 2‐Cys peroxiredoxins‐structures, mechanisms and functions. FEBS J 276: 2469–77. 19476488 10.1111/j.1742-4658.2009.06985.x PMC2747500

[43] Wood ZA , Poole LB , Karplus PA . 2003 Peroxiredoxin evolution and the regulation of hydrogen peroxide signaling. Science 300: 650–3. 12714747 10.1126/science.1080405

[44] Weerapana E , Wang C , Simon GM , Richter F , et al. 2010 Quantitative reactivity profiling predicts functional cysteines in proteomes. Nature 468: 790–5. 21085121 10.1038/nature09472 PMC3058684

[45] Rutter J , Reick M , Wu LC , McKnight SL . 2001 Regulation of clock and NPAS2 DNA binding by the redox state of NAD cofactors. Science 293: 510–4. 11441146 10.1126/science.1060698

[46] Wang TA , Yu YV , Govindaiah G , Ye X , et al. 2012 Circadian rhythm of redox state regulates excitability in suprachiasmatic nucleus neurons. Science 337: 839–42. 22859819 10.1126/science.1222826 PMC3490628

[47] Krishnan N , Davis AJ , Giebultowicz JM . 2008 Circadian regulation of response to oxidative stress in *Drosophila melanogaster* . Biochem Biophys Res Commun 374: 299–303. 18627767 10.1016/j.bbrc.2008.07.011 PMC2553425

[48] Lai AG , Doherty CJ , Mueller‐Roeber B , Kay SA , et al. 2012 CIRCADIAN CLOCK‐ASSOCIATED 1 regulates ROS homeostasis and oxidative stress responses. Proc Natl Acad Sci USA 109: 17129–34. 23027948 10.1073/pnas.1209148109 PMC3479464

[49] Wu L , Reddy AB . 2014 Rethinking the clockwork: redox cycles and non‐transcriptional control of circadian rhythms. Biochem Soc Trans 42: 1–0. 24450621 10.1042/BST20130169

[50] McDearmon EL , Patel KN , Ko CH , Walisser JA , et al. 2006 Dissecting the functions of the mammalian clock protein BMAL1 by tissue‐specific rescue in mice. Science 314: 1304–8. 17124323 10.1126/science.1132430 PMC3756687

[51] Mohawk JA , Baer ML , Menaker M . 2009 The methamphetamine‐sensitive circadian oscillator does not employ canonical clock genes. Proc Natl Acad Sci USA 106: 3519–24. 19204282 10.1073/pnas.0813366106 PMC2651344

[52] Wijnen H , Young MW . 2006 Interplay of circadian clocks and metabolic rhythms. Annu Rev Genet 40: 409–48. 17094740 10.1146/annurev.genet.40.110405.090603

[53] Eckel‐Mahan K , Sassone‐Corsi P . 2009 Metabolism control by the circadian clock and vice versa. Nat Struct Mol Biol 16: 462–7. 19421159 10.1038/nsmb.1595 PMC4073609

[54] Bass J . 2012 Circadian topology of metabolism. Nature 491: 348–56. 23151577 10.1038/nature11704

[55] Masri S , Sassone‐Corsi P . 2013 The circadian clock: a framework linking metabolism, epigenetics and neuronal function. Nat Rev Neurosci 14: 69–75. 23187814 10.1038/nrn3393 PMC5720680

[56] Bass J , Takahashi JS . 2010 Circadian integration of metabolism and energetics. Science 330: 1349–54. 21127246 10.1126/science.1195027 PMC3756146

[57] Rutter J , Reick M , McKnight SL . 2002 Metabolism and the control of circadian rhythms. Annu Rev Biochem 71: 307–31. 12045099 10.1146/annurev.biochem.71.090501.142857

[58] Nakahata Y , Kaluzova M , Grimaldi B , Sahar S , et al. 2008 The NAD+‐dependent deacetylase SIRT1 modulates CLOCK‐mediated chromatin remodeling and circadian control. Cell 134: 329–40. 18662547 10.1016/j.cell.2008.07.002 PMC3526943

[59] Asher G , Gatfield D , Stratmann M , Reinke H , et al. 2008 SIRT1 regulates circadian clock gene expression through PER2 deacetylation. Cell 134: 317–28. 18662546 10.1016/j.cell.2008.06.050

[60] Nakahata Y , Sahar S , Astarita G , Kaluzova M , et al. 2009 Circadian control of the NAD+ salvage pathway by CLOCK‐SI RT1. Science 324: 654–7. 19286518 10.1126/science.1170803 PMC6501775

[61] Sahar S , Masubuchi S , Eckel‐Mahan K , Vollmer S , et al. 2014 Circadian control of fatty acid elongation by SIRT1 protein‐mediated deacetylation of acetyl‐coenzyme A synthetase 1. J Biol Chem 289: 6091–7. 24425865 10.1074/jbc.M113.537191 PMC3937675

[62] Ramsey KM , Yoshino J , Brace CS , Abrassart D , et al. 2009 Circadian clock feedback cycle through NAMPT‐mediated NAD+ biosynthesis. Science 324: 651–4. 19299583 10.1126/science.1171641 PMC2738420

[63] Asher G , Reinke H , Altmeyer M , Gutierrez‐Arcelus M , et al. 2010 Poly(ADP‐ribose) polymerase 1 participates in the phase entrainment of circadian clocks to feeding. Cell 142: 943–53. 20832105 10.1016/j.cell.2010.08.016

[64] Peek CB , Affinati AH , Ramsey KM , Kuo HY , et al. 2013 Circadian clock NAD+ cycle drives mitochondrial oxidative metabolism in mice. Science 342: 1243417. 24051248 10.1126/science.1243417 PMC3963134

[65] Asher G , Schibler U . 2011 Crosstalk between components of circadian and metabolic cycles in mammals. Cell Metab 13: 125–37. 21284980 10.1016/j.cmet.2011.01.006

[66] Ghosh A , Chance B . 1964 Oscillations of glycolytic intermediates in yeast cells. Biochem Biophys Res Commun 16: 174–81. 4224521 10.1016/0006-291x(64)90357-2

[67] Bier M , Bakker BM , Westerhoff HV . 2000 How yeast cells synchronize their glycolytic oscillations: a perturbation analytic treatment. Biophys J 78: 1087–93. 10692299 10.1016/S0006-3495(00)76667-7 PMC1300712

[68] Kloster A , Olsen LF . 2012 Oscillations in glycolysis in *Saccharomyces cerevisiae*: the role of autocatalysis and intracellular ATPase activity. Biophys Chem 165‐166: 39–47. 10.1016/j.bpc.2012.03.003 22459703

[69] Zhang EE , Liu Y , Dentin R , Pongsawakul PY , et al. 2010 Cryptochrome mediates circadian regulation of cAMP signaling and hepatic gluconeogenesis. Nat Med 16: 1152–6. 20852621 10.1038/nm.2214 PMC2952072

[70] Akimoto H , Kinumi T , Ohmiya Y . 2005 Circadian rhythm of a TCA cycle enzyme is apparently regulated at the translational level in the dinoflagellate *Lingulodinium polyedrum* . J Biol Rhythms 20: 479–89. 16275767 10.1177/0748730405280811

[71] Guo J , Nguyen AY , Dai Z , Su D , et al. 2014 Proteome‐wide light/dark modulation of thiol oxidation in cyanobacteria revealed by quantitative site‐specific redox proteomics. Mol Cell Proteomics 13: 3270–85. 25118246 10.1074/mcp.M114.041160 PMC4256482

[72] Masri S , Patel VR , Eckel‐Mahan KL , Peleg S , et al. 2013 Circadian acetylome reveals regulation of mitochondrial metabolic pathways. Proc Natl Acad Sci USA 110: 3339–44. 23341599 10.1073/pnas.1217632110 PMC3587221

[73] Dallmann R , Viola AU , Tarokh L , Cajochen C , et al. 2012 The human circadian metabolome. Proc Natl Acad Sci USA 109: 2625–9. 22308371 10.1073/pnas.1114410109 PMC3289302

[74] Davies SK , Ang JE , Revell VL , Holmes B , et al. 2014 Effect of sleep deprivation on the human metabolome. Proc Natl Acad Sci USA 111: 10761–6. 25002497 10.1073/pnas.1402663111 PMC4115565

[75] Martinez‐Lozano SP , Tarokh L , Li X , Kohler M , et al. 2014 Circadian variation of the human metabolome captured by real‐time breath analysis. PLoS ONE 9: e114422. 25545545 10.1371/journal.pone.0114422 PMC4278702

[76] Yamazaki S , Numano R , Abe M , Hida A , et al. 2000 Resetting central and peripheral circadian oscillators in transgenic rats. Science 288: 682–5. 10784453 10.1126/science.288.5466.682

[77] Reppert SM , Weaver DR . 2001 Molecular analysis of mammalian circadian rhythms. Annu Rev Physiol 63: 647–76. 11181971 10.1146/annurev.physiol.63.1.647

[78] Fuller PM , Gooley JJ , Saper CB . 2006 Neurobiology of the sleep‐wake cycle: sleep architecture, circadian regulation, and regulatory feedback. J Biol Rhythms 21: 482–93. 17107938 10.1177/0748730406294627

[79] Deboer T , Vansteensel MJ , Detari L , Meijer JH . 2003 Sleep states alter activity of suprachiasmatic nucleus neurons. Nat Neurosci 6: 1086–90. 12958601 10.1038/nn1122

[80] Borbely AA . 1982 A two process model of sleep regulation. Hum Neurobiol 1: 195–204. 7185792

[81] Franken P , Dijk DJ . 2009 Circadian clock genes and sleep homeostasis. Eur J Neurosci 29: 1820–9. 19473235 10.1111/j.1460-9568.2009.06723.x

[82] Franken P . 2013 A role for clock genes in sleep homeostasis. Curr Opin Neurobiol 23: 864–72. 23756047 10.1016/j.conb.2013.05.002

[83] Shaw PJ , Tononi G , Greenspan RJ , Robinson DF . 2002 Stress response genes protect against lethal effects of sleep deprivation in Drosophila. Nature 417: 287–91. 12015603 10.1038/417287a

[84] Wisor JP , Pasumarthi RK , Gerashchenko D , Thompson CL , et al. 2008 Sleep deprivation effects on circadian clock gene expression in the cerebral cortex parallel electroencephalographic differences among mouse strains. J Neurosci 28: 7193–201. 18614689 10.1523/JNEUROSCI.1150-08.2008 PMC2603080

[85] Krueger JM , Obal F, Jr . 2003 Sleep function. Front Biosci 8: d511–9. 12700033 10.2741/1031

[86] Huber R , Ghilardi MF , Massimini M , Tononi G . 2004 Local sleep and learning. Nature 430: 78–81. 15184907 10.1038/nature02663

[87] Ferrara M , De GL . 2011 Going local: insights from EEG and stereo‐EEG studies of the human sleep‐wake cycle. Curr Top Med Chem 11: 2423–37. 21906022 10.2174/156802611797470268

[88] Vyazovskiy VV , Olcese U , Hanlon EC , Nir Y , et al. 2011 Local sleep in awake rats. Nature 472: 443–7. 21525926 10.1038/nature10009 PMC3085007

[89] Krueger JM , Rector DM , Roy S , Van Dongen HP , et al. 2008 Sleep as a fundamental property of neuronal assemblies. Nat Rev Neurosci 9: 910–9. 18985047 10.1038/nrn2521 PMC2586424

[90] Hinard V , Mikhail C , Pradervand S , Curie T , et al. 2012 Key electrophysiological, molecular, and metabolic signatures of sleep and wakefulness revealed in primary cortical cultures. J Neurosci 32: 12506–17. 22956841 10.1523/JNEUROSCI.2306-12.2012 PMC6621272

[91] Cirelli C , Gutierrez CM , Tononi G . 2004 Extensive and divergent effects of sleep and wakefulness on brain gene expression. Neuron 41: 35–43. 14715133 10.1016/s0896-6273(03)00814-6

[92] Robles MS , Mann M . 2013 Proteomic approaches in circadian biology. Handb Exp Pharmacol 2013: 389–407. 23604489 10.1007/978-3-642-25950-0_17

[93] Moller M , Sparre T , Bache N , Roepstorff P , et al. 2007 Proteomic analysis of day‐night variations in protein levels in the rat pineal gland. Proteomics 7: 2009–18. 17514675 10.1002/pmic.200600963

[94] Deery MJ , Maywood ES , Chesham JE , Sladek M , et al. 2009 Proteomic analysis reveals the role of synaptic vesicle cycling in sustaining the suprachiasmatic circadian clock. Curr Biol 19: 2031–6. 19913422 10.1016/j.cub.2009.10.024

[95] Ong SE , Mann M . 2005 Mass spectrometry‐based proteomics turns quantitative. Nat Chem Biol 1: 252–62. 16408053 10.1038/nchembio736

[96] Lee JE , Zamdborg L , Southey BR , Atkin N, Jr. , et al. 2013 Quantitative peptidomics for discovery of circadian‐related peptides from the rat suprachiasmatic nucleus. J Proteome Res 12: 585–93. 23256577 10.1021/pr300605p PMC3562399

[97] Lee JE , Atkins N, Jr. , Hatcher NG , Zamdborg L , et al. 2010 Endogenous peptide discovery of the rat circadian clock: a focused study of the suprachiasmatic nucleus by ultrahigh performance tandem mass spectrometry. Mol Cell Proteomics 9: 285–97. 19955084 10.1074/mcp.M900362-MCP200 PMC2830840

[98] Seo HS , Hirano M , Shibato J , Rakwal R , et al. 2008 Effects of coffee bean aroma on the rat brain stressed by sleep deprivation: a selected transcript‐ and 2D gel‐based proteome analysis. J Agric Food Chem 56: 4665–73. 18517217 10.1021/jf8001137

[99] Pawlyk AC , Ferber M , Shah A , Pack AI , et al. 2007 Proteomic analysis of the effects and interactions of sleep deprivation and aging in mouse cerebral cortex. J Neurochem 103: 2301–13. 17919293 10.1111/j.1471-4159.2007.04949.x

[100] Janich P , Arpat AB , Castelo‐Szekely V , Lopes M , et al. 2015 Ribosome profiling reveals the rhythmic liver translatome and circadian clock regulation by upstream open reading frames. Genome Res 25: 1848–59. 26486724 10.1101/gr.195404.115 PMC4665006

[101] Jang C , Lahens NF , Hogenesch JB , Sehgal A . 2015 Ribosome profiling reveals an important role for translational control in circadian gene expression. Genome Res 25: 1836–47. 26338483 10.1101/gr.191296.115 PMC4665005

[102] Zhou M , Wang W , Karapetyan S , Mwimba M , et al. 2015 Redox rhythm reinforces the circadian clock to gate immune response. Nature 523: 472–6. 26098366 10.1038/nature14449 PMC4526266

[103] Yoshida Y , Iigusa H , Wang N , Hasunuma K . 2011 Cross‐talk between the cellular redox state and the circadian system in Neurospora. PLoS ONE 6: e28227. 22164247 10.1371/journal.pone.0028227 PMC3229512

[104] Gillette MU , Wang TA . 2014 Brain circadian oscillators and redox regulation in mammals. Antioxid Redox Signal 20: 2955–65. 24111727 10.1089/ars.2013.5598 PMC4038987

[105] Mann M , Jensen ON . 2003 Proteomic analysis of post‐translational modifications. Nat Biotechnol 21: 255–61. 12610572 10.1038/nbt0303-255

[106] Kim EY , Edery I . 2006 Balance between DBT/CKIepsilon kinase and protein phosphatase activities regulate phosphorylation and stability of Drosophila CLOCK protein. Proc Natl Acad Sci USA 103: 6178–83. 16603629 10.1073/pnas.0511215103 PMC1458851

[107] Hirano A , Yumimoto K , Tsunematsu R , Matsumoto M , et al. 2013 FBXL21 regulates oscillation of the circadian clock through ubiquitination and stabilization of cryptochromes. Cell 152: 1106–18. 23452856 10.1016/j.cell.2013.01.054

[108] Cardone L , Hirayama J , Giordano F , Tamaru T , et al. 2005 Circadian clock control by SUMOylation of BM AL1. Science 309: 1390–4. 16109848 10.1126/science.1110689

[109] Eckel‐Mahan KL , Patel VR , Mohney RP , Vignola KS , et al. 2012 Coordination of the transcriptome and metabolome by the circadian clock. Proc Natl Acad Sci USA 109: 5541–6. 22431615 10.1073/pnas.1118726109 PMC3325727

[110] Patel VR , Eckel‐Mahan K , Sassone‐Corsi P , Baldi P . 2012 CircadiOmics: integrating circadian genomics, transcriptomics, proteomics, and metabolomics. Nat Methods 9: 772–3. 22847108 10.1038/nmeth.2111

[111] Zhang R , Lahens NF , Ballance HI , Hughes ME , et al. 2014 A circadian gene expression atlas in mammals: implications for biology and medicine. Proc Natl Acad Sci USA 111: 16219–24. 25349387 10.1073/pnas.1408886111 PMC4234565

[112] Petit JM , Burlet‐Godinot S , Magistretti PJ , Allaman I . 2015 Glycogen metabolism and the homeostatic regulation of sleep. Metab Brain Dis 30: 263–79. 25399336 10.1007/s11011-014-9629-x PMC4544655

[113] Benington JH , Heller HC . 1995 Restoration of brain energy metabolism as the function of sleep. Prog Neurobiol 45: 347–60. 7624482 10.1016/0301-0082(94)00057-o

[114] Van CE , Spiegel K , Tasali E , Leproult R . 2008 Metabolic consequences of sleep and sleep loss. Sleep Med 9: S23–8. 18929315 10.1016/S1389-9457(08)70013-3 PMC4444051

[115] Grimaldi B , Bellet MM , Katada S , Astarita G , et al. 2010 PER2 controls lipid metabolism by direct regulation of PPARgamma. Cell Metab 12: 509–20. 21035761 10.1016/j.cmet.2010.10.005 PMC4103168

[116] Van der Horst GT , Muijtjens M , Kobayashi K , Takano R , et al. 1999 Mammalian Cry1 and Cry2 are essential for maintenance of circadian rhythms. Nature 398: 627–30. 10217146 10.1038/19323

[117] Zaniolo K , Desnoyers S , Leclerc S , Guerin SL . 2007 Regulation of poly(ADP‐ribose) polymerase‐1 (PARP‐1) gene expression through the post‐translational modification of Sp1: a nuclear target protein of PARP‐1. BMC Mol Biol 8: 96. 17961220 10.1186/1471-2199-8-96 PMC2175517

[118] Hallows WC , Lee S , Denu JM . 2006 Sirtuins deacetylate and activate mammalian acetyl‐CoA synthetases. Proc Natl Acad Sci USA 103: 10230–5. 16790548 10.1073/pnas.0604392103 PMC1480596

[119] Lamia KA , Sachdeva UM , DiTacchio L , Williams EC , et al. 2009 AMPK regulates the circadian clock by cryptochrome phosphorylation and degradation. Science 326: 437–40. 19833968 10.1126/science.1172156 PMC2819106

[120] Liu C , Li S , Liu T , Borjigin J , et al. 2007 Transcriptional coactivator PGC‐1alpha integrates the mammalian clock and energy metabolism. Nature 447: 477–81. 17476214 10.1038/nature05767

[121] Rodgers JT , Lerin C , Haas W , Gygi SP , et al. 2005 Nutrient control of glucose homeostasis through a complex of PGC‐1alpha and SIRT1. Nature 434: 113–8. 15744310 10.1038/nature03354

[122] Kaasik K , Lee CC . 2004 Reciprocal regulation of haem biosynthesis and the circadian clock in mammals. Nature 430: 467–71. 15269772 10.1038/nature02724

[123] Reinke H , Saini C , Fleury‐Olela F , Dibner C , et al. 2008 Differential display of DNA‐binding proteins reveals heat‐shock factor 1 as a circadian transcription factor. Genes Dev 22: 331–45. 18245447 10.1101/gad.453808 PMC2216693

[124] O'Neill JS , Maywood ES , Chesham JE , Takahashi JS , et al. 2008 CAMP‐dependent signaling as a core component of the mammalian circadian pacemaker. Science 320: 949–53. 18487196 10.1126/science.1152506 PMC2735813

[125] Lee B , Li A , Hansen KF , Cao R , et al. 2010 CREB influences timing and entrainment of the SCN circadian clock. J Biol Rhythms 25: 410–20. 21135157 10.1177/0748730410381229 PMC3529591

[126] Brunet A , Sweeney LB , Sturgill JF , Chua KF , et al. 2004 Stress‐dependent regulation of FOXO transcription factors by the SIRT1 deacetylase. Science 303: 2011–5. 14976264 10.1126/science.1094637

[127] Frescas D , Valenti L , Accili D . 2005 Nuclear trapping of the forkhead transcription factor FoxO1 via Sirt‐dependent deacetylation promotes expression of glucogenetic genes. J Biol Chem 280: 20589–95. 15788402 10.1074/jbc.M412357200

[128] Sato TK , Panda S , Miraglia LJ , Reyes TM , et al. 2004 A functional genomics strategy reveals Rora as a component of the mammalian circadian clock. Neuron 43: 527–37. 15312651 10.1016/j.neuron.2004.07.018

[129] Schmutz I , Ripperger JA , Baeriswyl‐Aebischer S , Albrecht U . 2010 The mammalian clock component PERIOD2 coordinates circadian output by interaction with nuclear receptors. Genes Dev 24: 345–57. 20159955 10.1101/gad.564110 PMC2816734

